# A modular Golden Gate toolkit for *Yarrowia lipolytica* synthetic biology

**DOI:** 10.1111/1751-7915.13427

**Published:** 2019-05-31

**Authors:** Macarena Larroude, Young‐Kyoung Park, Paul Soudier, Monika Kubiak, Jean‐Marc Nicaud, Tristan Rossignol

**Affiliations:** ^1^ Micalis Institute AgroParisTech INRA Université Paris‐Saclay 78350 Jouy‐en‐Josas France; ^2^ Department of Biotechnology and Food Microbiology Poznan University of Life Sciences ul. Wojska Polskiego 48 60‐627 Poznan Poland

## Abstract

The oleaginous yeast *Yarrowia lipolytica* is an established host for the bio‐based production of valuable compounds and an organism for which many genetic tools have been developed. However, to properly engineer *Y*.* lipolytica* and take full advantage of its potential, we need efficient, versatile, standardized and modular cloning tools. Here, we present a new modular Golden Gate toolkit for the one‐step assembly of three transcription units that includes a selective marker and sequences for genome integration. Perfectly suited to a combinatorial approach, it contains nine different validated promoters, including inducible promoters, which allows expression to be fine‐tuned. Moreover, this toolbox incorporates six different markers (three auxotrophic markers, two antibiotic‐resistance markers and one metabolic marker), which allows the fast sequential construction and transformation of multiple elements. In total, the toolbox contains 64 bricks, and it has been validated and characterized using three different fluorescent reporter proteins. Additionally, it was successfully used to assemble and integrate a three‐gene pathway allowing xylose utilization by *Y. lipolytica*. This toolbox provides a powerful new tool for rapidly engineering *Y. lipolytica* strains and is available to the community through Addgene.

## Introduction

*Yarrowia lipolytica* is the most well‐developed and well‐researched yeast in the domain of oleochemical production (Beopoulos *et al*., 2009; Ledesma‐Amaro and Nicaud, 2016; Lazar *et al*., 2018). It is considered to be a GRAS organism (Groenewald *et al*., 2013) and has an established history within the biotechnology industry. It has been intensively used for various applications, ranging from biofuel to vaccine production (Madzak, 2018). However, when the goal is to produce a compound at the industrial scale, multiple rounds of metabolic engineering are usually needed. For example, for *Y. lipolytica* to produce omega‐3 eicosapentaenoic acid at industrial levels, it has been necessary to integrate up to 30 copies of nine different genes and carry out one deletion (Xue *et al*., 2013). Similarly, laboratory‐scale production of ricinoleic acid has required the overexpression of three genes and the deletion of up to 10 genes, mainly due to the redundancy of genes involved in lipid metabolism in oleaginous microorganisms (Beopoulos *et al*., 2014). Therefore, when seeking to re‐engineer such specialized microorganisms, it can be extremely tedious, time‐consuming and cost‐ineffective to work with the existing genetic background. It requires massive efforts to integrate an equivalent series of modifications into a new wild‐type strain that presents specific or more appropriate traits with a view to creating new producer or chassis strains. Moreover, strain reconstruction requires multiple steps that sometimes lead to a final construct that does not display the expected phenotype or production yield because the transformation process results in the accumulation of potential trade‐offs resulting from random insertions for example.

Moreover, to obtain an optimized producer strain using the design–build–test–learn cycle, it is necessary to perform large‐scale screening using the combinatorial expression of pathway components and to fine‐tune control of gene expression. Rapid, efficient and combinatorial cloning tools are needed for such approaches.

For these reasons, researchers are developing versatile, standardized and modular tools for carrying out genetic engineering and genome editing in *Y. lipolytica*, as such tools have recently become available in the model yeast *Saccharomyces cerevisiae* (Lee *et al*., 2015). Two key tools have recently been released for *Y. lipolytica*: EasyCloneYALI, which is based on an alternative USER cloning approach (Holkenbrink *et al*., 2018), and the YaliBricks system (Wong *et al*., 2017), which utilizes the BioBricks standard and four compatible restriction enzyme sites. Here, we present the development and release of a new modular toolkit dedicated to *Y. lipolytica* that is based on the popular Golden Gate (GG) strategy (Engler *et al*., 2009). It allows the one‐step assembly of up to three transcription units (TUs) together with a selective marker and sequences for random or targeted genome integration. The toolkit has been designed for optimum modularity and flexibility: it makes available nine promoters of varying strengths, including inducible promoters that are perfectly suited to industrial applications. All the promoters are available at each TU position. The toolkit also includes five terminators, six selection markers (three auxotrophic markers, two antibiotic‐resistance markers and one metabolic marker) and four pairs of bricks for random or targeted genome integration. A detailed description is provided of GG brick design and assembly (see supplementary protocol S4). All the bricks are available to the community through Addgene. Our toolkit is a powerful new tool for rapidly engineering *Y. lipolytica* strains.

## Results

### Brick building

**Figure 1:**
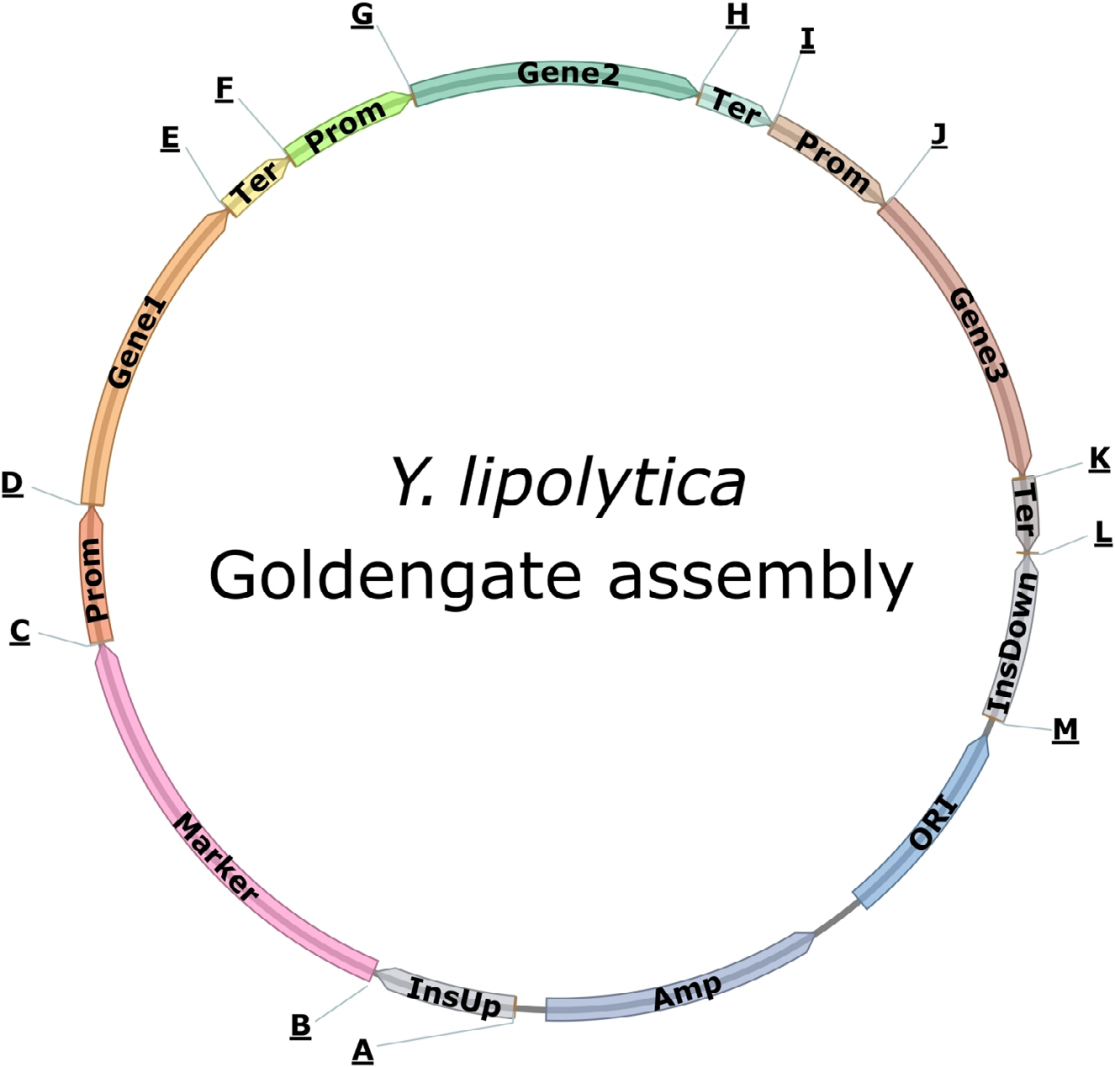
Schematic drawing of the 3‐TU GG assembly with letters indicating the 4‐nt overhang flanking each GG brick. The sequences of each 4‐nt overhang are available in Fig. S1. InsUp and InsDown: sequences for insertion in the genome; Prom: promoter; Ter: terminator.

The GG strategy used in our *Y. lipolytica* toolkit utilizes the BsaI Type IIS restriction enzyme. Thirteen unique overhang sequences were designed that allow the oriented assembly of up to three TUs together with a selection marker, upstream and downstream sequences for random or targeted genome insertion, and the *Escherichia coli* replicative backbone. Each TU is composed of a promoter, a gene of interest and a terminator. An assembled vector is depicted in Fig. 1; the letters indicate the four nucleotide overhang sequences (described in Fig. S1.)

#### Promoters

To fine‐tune and allow combinatorial expression of each TU in this assembly system, promoters with a broad range of strengths must be implemented. Therefore, six promoters that displayed weak to very strong expression strength were constructed. There were three native promoters: the widely used p*TEF* promoter and two other lower‐strength promoters, p*PGM* and p*GAP* (Larroude *et al*., 2018). There were also three synthetic hybrid promoters, which were composed of a core promoter preceded by two to eight repeated UAS sequences (Dulermo *et al*., 2017). In addition, we included three hybrid promoters that are inducible by erythritol. They are based on the recently developed p*EYK1* promoter and incorporate three to five repeated UAS sequences (Trassaert *et al*., 2017; Park *et al*., 2019). Consequently, in total, there are nine promoters available for the three TUs.

**Figure 2:**
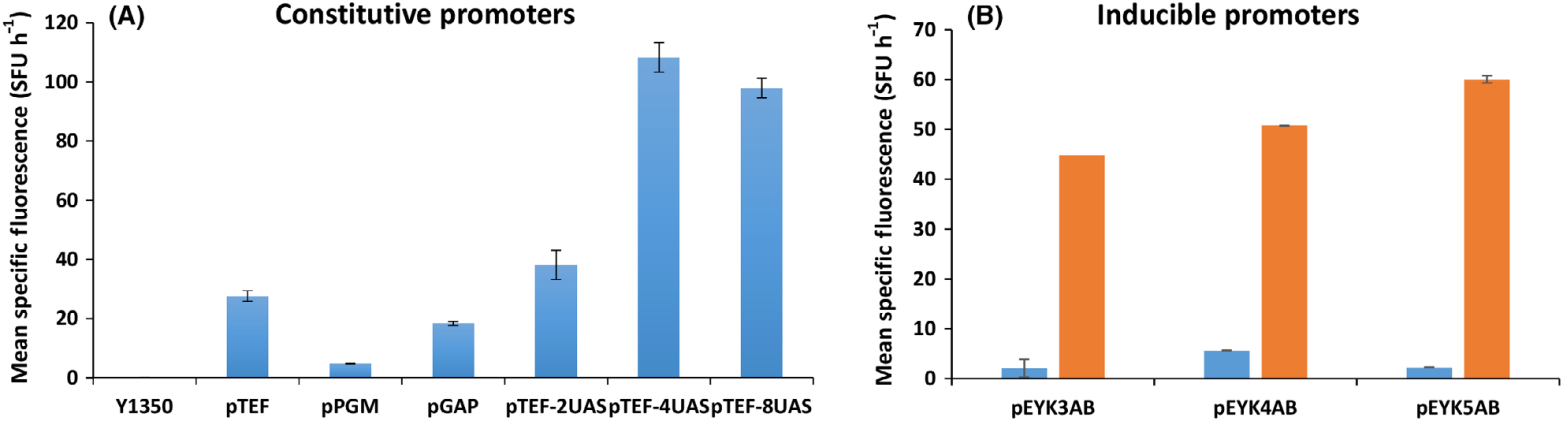
Expression associated with the nine promoters using *RedStarII* as a reporter gene in position 1 (first TU). A. Constitutive promoters, using glucose as the carbon source. B. Inducible promoters, using glucose (blue bars) and erythritol (orange bars) as the carbon sources. Promoters were tested on the strain JMY1212, and a construct‐free strain (JMY1350; Table S1) was the control. The values correspond to the mean of two independent clones that randomly integrated each construct. Error bars represent standard deviations.

Figure 2 shows the expression levels associated with the nine promoters when RedStarII was employed as the reporter gene in position 1 (first TU); glucose was the carbon source for the constitutive promoters (Fig. 2A), and glucose and erythritol were the carbon sources for the inducible promoters (Fig. 2B). Under these conditions, expression levels were lower with p*PGM* and p*GAP* than with p*TEF*. In contrast, expression levels were relatively higher with all the synthetic promoters, and the promoters with four and eight UASs performed better than the one with two UASs. The 8‐UAS promoter and the 4‐UAS promoter resulted in similar expression levels and thus displayed similar performance in this condition. Either we achieved maximum expression under our conditions with 4UAS p*TEF* and 8UAS p*TEF* or the latter was not the best suited promoter for the RedStarII under these conditions (Dulermo *et al*., 2017). For the inducible promoters, expression level was positively correlated with UAS1‐eyk1 copy number when the medium contained erythritol; in contrast, expression level remained very low when the medium contained only glucose, as previously reported (Park *et al*., 2019).

#### Terminators

**Figure 3:**
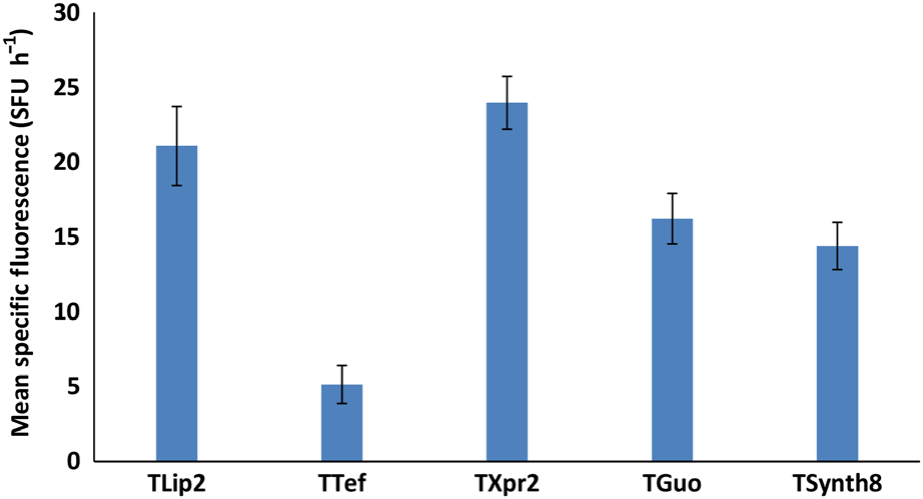
Impact of different terminators on gene expression levels using *RedStarII* as a reporter gene under the p*TEF* promoter. The bars correspond to mean specific red fluorescence (position 1). The values correspond to the mean of five to seven independent clones that randomly integrated each construction. JMY1212 strain was used. Error bars represent standard deviations.

Terminators are major components of expression systems because they are responsible for terminating transcription and because they play a role in mRNA half‐life. In our toolkit, we have included five different terminators (Celinska *et al*., 2017) (Fig. S1). To characterize their impact on expression level, all the terminators were tested in position 1 (first TU) using the p*TEF* promoter and the *RedStarII* reporter gene. We found that each one allowed the correct expression of the reporter gene, but there were differences in expression strength: T*Lip2* resulted in the greatest expression, and T*Tef* resulted in the lowest expression (Fig. 3). The role of terminators in mediating expression levels has been extensively described in *Saccharomyces cerevisiae* (Curran *et al*., 2015; Redden *et al*., 2015) and somewhat characterized in *Y. lipolytica* (Curran *et al*., 2015). The terminators T1*Guo* and T*Synth8* resulted in similar levels of expression, as reported previously (Curran *et al*., 2015). In this study, however, expression levels were lower with T*Tef* than with T1*Guo* and T*Synth8*, while the opposite was true in Curran *et al*. (2015). This discrepancy can be explained by the fact that the latter study used a different expression cassette, one that bore the *HrGFP* reporter gene and a hybrid promoter. More importantly, the terminator sequences were longer than the ones used here (253 base pairs versus 66 base pairs). As a result, this toolkit allows expression to be fine‐tuned using various combinations of terminators and promoters. Furthermore, this approach helps limit the occurrence of homologous sequences inside the vector and thus reduces the risk of recombination and the loss of part of the constructs. To allow the assembly of just one or two TUs, we have also provided T*Lip2* terminators containing the BsaI overhang sequences E and L and terminators containing the BsaI overhang sequences H and L; the result is that there is no need to change the overhang sequences of the brick genes (Fig. S1).

#### Markers

The core principle of the GG strategy is modularity and versatility. Consequently, our set of bricks includes a panel of markers that meet the requirements of most *Y. lipolytica* genetic backgrounds. We implemented three auxotrophic markers, *URA3*,* LEU2* and *LYS5*, which are the most popular auxotrophic markers used with laboratory strains. Two markers for antibiotic resistance (against hygromycin and nourseothricin respectively) are also available; they can be used with auxotrophic strains as well as with wild‐type strains. In addition, the toolkit includes a metabolic marker, the invertase from *S. cerevisiae*, which allows growth on sucrose (Lazar *et al*., 2013). This large panel of markers makes it possible to carry out numerous successive transformations without the need to recycle markers. Moreover, the latter three markers can be used to modify wild‐type strains. All the markers were successfully used in the assembly process with a RedStarII expression cassette (Table S1) and were transformed into an appropriate background for validation (data not shown).

#### Integration sites

To extend the genetic engineering capabilities of our system, we have included several flanking sequences that allow integration at either a random locus or a specific locus. ZETA sequences are widely used and allow random integration; however, they also allow targeted integration into the zeta‐docking platform when it is present in strains. Such strains are employed in various bioprocesses (e.g. JMY1212; Bordes *et al*., 2007) or are used to carry out the large‐scale screening of homologous or heterologous gene overexpression (e.g. JMY2566; Leplat *et al*., 2015; Beneyton *et al*., 2017). When using ZETA sequences, the presence of the docking platform strongly favours integration, with success rates of up to 84% (Bordes *et al*., 2007).

**Figure 4:**
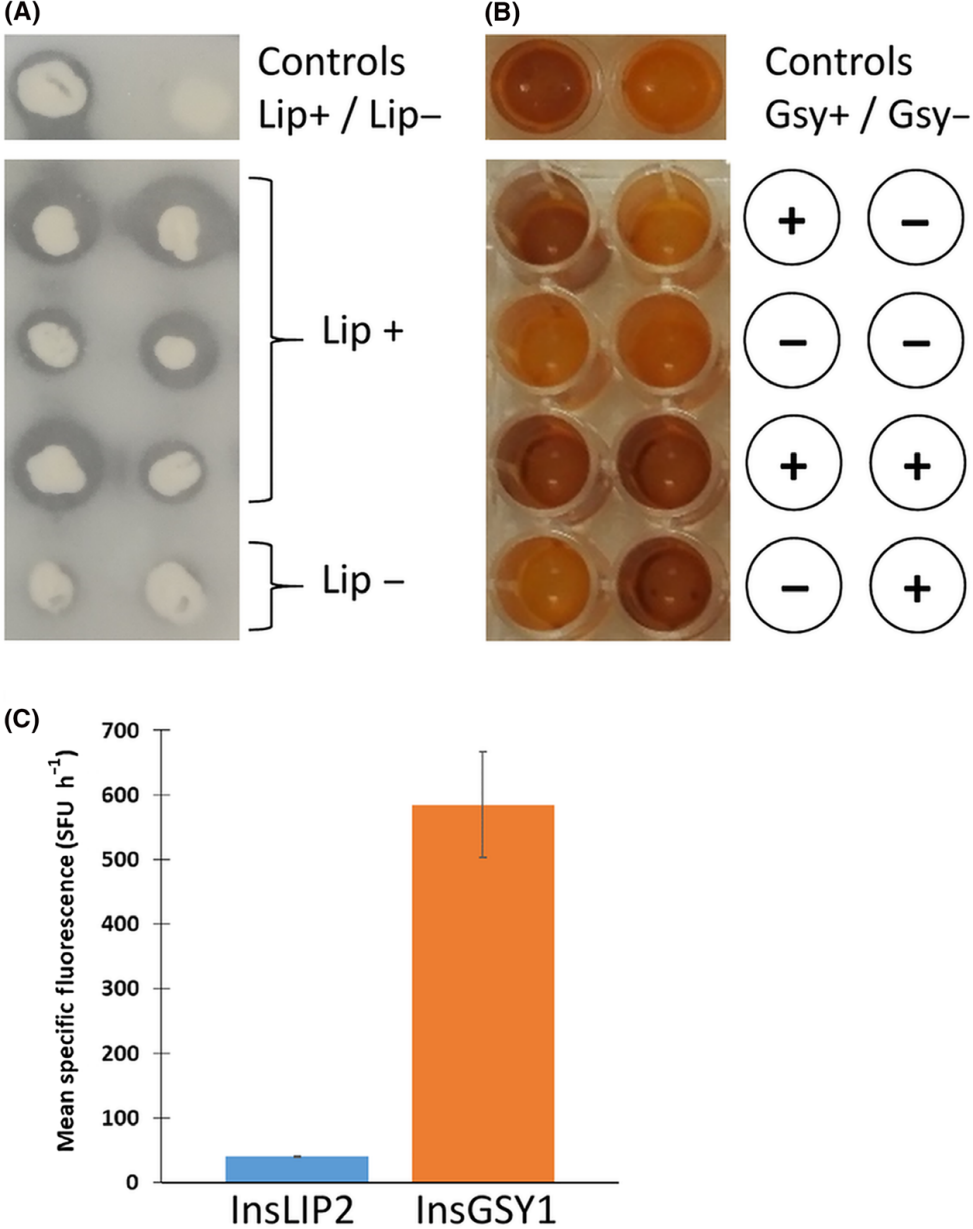
Examples of successful integration at the locus sites. A. Detection of lipase activity on a tributyrin plate. When the colonies were surrounded by a clear zone, they were capable of lipase production. The GGA was used to transform a JMY195 strain, and the results were compared with those for the wild type, W29, and the lipase defective strain, JMY1212. B. Detection of glycogen synthase activity using Lugol's solution. When yellow wells were present, it indicated a defect in glycogen synthase, which is a characteristic of the Δgsy1 mutant strain (Bhutada *et al*., 2017). In this case, the JMY1212 strain was used to test the integration at gsy locus. These results were compared with those for the wild type, W29. C. Impact of integration site on gene expression levels using *RedStarII* as a reporter gene. The bars correspond to mean specific red fluorescence. The values correspond to the mean of four independent clones that have a correct locus integration. Error bars represent standard deviations.

In addition, there is an industrial interest in deleting certain genes, namely *LIP2*,* GSY1* or *MFE*. Consequently, their promoter and terminator regions were added to the list of insertion sites. This approach allows an expression cassette of interest to be integrated while simultaneously deleting a gene or pathway that interferes with biotechnological applications in the domain of lipid metabolism. Moreover, we choose targets for which phenotype screening is easier: the halo is reduced on plates containing tributyrin in the case of *lip2∆* strains (Pignede *et al*., 2000); cells appear less brown when exposed to Lugol's solution in the case of *gsy1∆* strains (Bhutada *et al*., 2017); and cells cannot grow on media containing lipids as the only carbon source in the case of *mfe∆* strains. We evaluated the integration of a RedStarII expression cassette under a p*TEF* promoter flanked by LIP2 or GSY1 insertion site sequences. For LIP2, among the 37 transformants that showed red fluorescence, five displayed the *Δlip2* phenotype on a tributyrin plate (see the example in Fig. 4A) and reflected proper integration at the lip2 locus via homologous recombination, which corresponds to a 14% integration rate. For GSY1, among the 24 transformants that showed red fluorescence, 11 displayed the *Δgsy1* phenotype after Lugol's iodine staining (see the example in Fig. 4B) and reflected proper integration at the gsy1 locus via homologous recombination, which corresponds to a 45% integration rate. The integration rate at the lip2 locus was unsurprising because *Y. lipolytica* has a low homologous recombination efficiency. In contrast, we have always obtained higher recombination rates for the gsy1 locus (data not shown). The RedStarII fluorescence level is also much higher when expressed at the gsy1 locus compared with the lip2 locus (Fig. 4C). This latter shows similar level to random integration.

Users can easily build upon this list, by adding flanking sequences of their own that target specific DNA regions; we provide a detailed protocol in the Supplementary material.

#### Destination vector

The two destination vector backbones provided in this GG toolkit contain the red fluorescence protein (RFP) chromophore, which acts as a colour‐based visual marker for negative cloning in *E. coli*, as described elsewhere (Celinska *et al*., 2017). The vector on GGE029 only contains the ampicillin resistance, the ColE1 region for selection and propagation in *E. coli* and the RFP flanked with the BsaI sites required (A and M) to assemble the expression cassettes. The vector on GGE114 is a preassembled destination vector that, in addition to the bacterial part, contains popular bricks – ZETA sequences in the place of InsUp and InsDown fragments and the *URA3* marker – with a view to reducing the number of fragments to assembly when employing this combination, which is the most common (Park *et al*., 2019). In this case, the RFP is between the *URA3* marker and the ZETA down. In the presence of BsaI, the RFP is released and the TU can be added in that place. Thus, both destination vectors can be used for assembling 1, 2 or 3 TU.

### Protein expression

The capacity and efficacy of our system to express three TUs on a same construct has been validated previously (Celinska *et al*., 2017; Larroude *et al*., 2018). However, the expression levels of the three TUs have not been evaluated. Here, we used three fluorescent proteins as reporters to validate and quantify TU expression levels.

#### Expression of the three fluorescent reporter proteins

**Figure 5:**
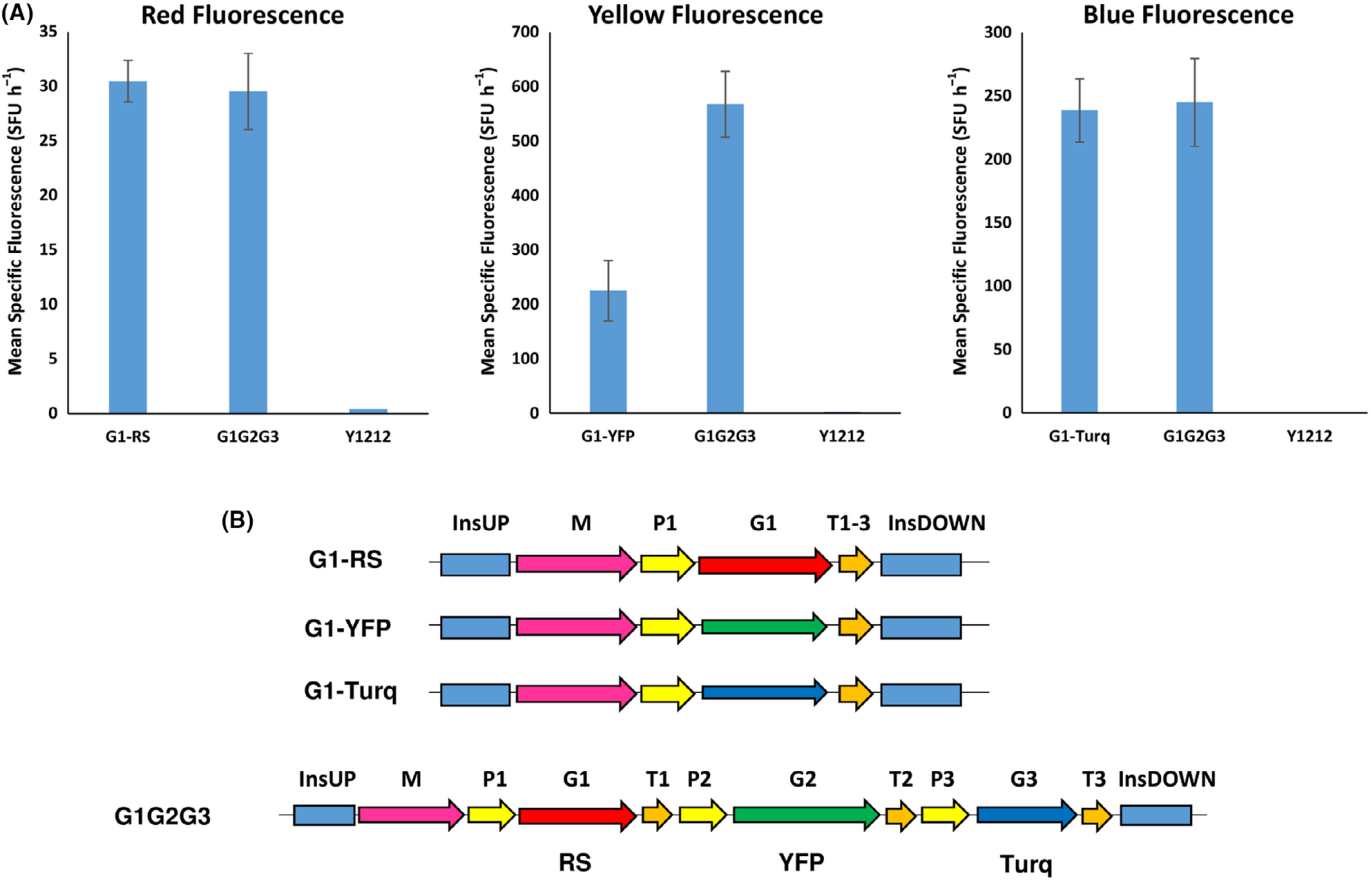
A. Mean specific fluorescence of strains expressing a single fluorescent protein – RedStarII, YFP or mTurquoise – in position 1 of a 1‐TU GG vector or of strains expressing all three fluorescent proteins in a 3‐TU GG vector. In G1G2G3, *RedStarII* is in position 1, *YFP* is in position 2, and *mTurquoise* is in position 3. JMY1212 was the control strain. The values correspond to the average for 8–10 independent clones that randomly integrated each construct. Error bars represent standard deviations. B. Schematic of the construct used, all of them were transformed into JMY1212 strain.

First, we evaluated the expression and detection of the three fluorescent proteins – RedStarII, YFP and mTurquoise – in position 1 in the *Y. lipolytica* GG system using p*TEF* and T*Lip2*. The fluorescence observed at these three wavelengths shows that the three proteins were correctly expressed (Fig. 5).

To evaluate expression levels at the different positions associated with the three TUs, the three proteins were assembled together in the same 3‐TU vector and using the same promoter (p*TEF*) and terminator (T*Lip2*) as in the above, single‐position experiment. RedStarII occurred at position 1, YFP occurred at position 2, and mTurquoise occurred at position 3. The three proteins were correctly expressed (Fig. 5). The expression levels of RedStarII and mTurquoise were similar to those obtained in the single‐position experiment; in contrast, the expression level of YFP was higher. We then switched around the positions of the fluorescent proteins. RedStarII and mTurquoise expression was unaffected by position; YFP behaved differently but only when it was in position 2 (data not shown). This finding reveals that, for some proteins, expression levels could be TU position‐dependent.

#### Assembly and expression of the xylose utilization pathway

**Figure 6:**
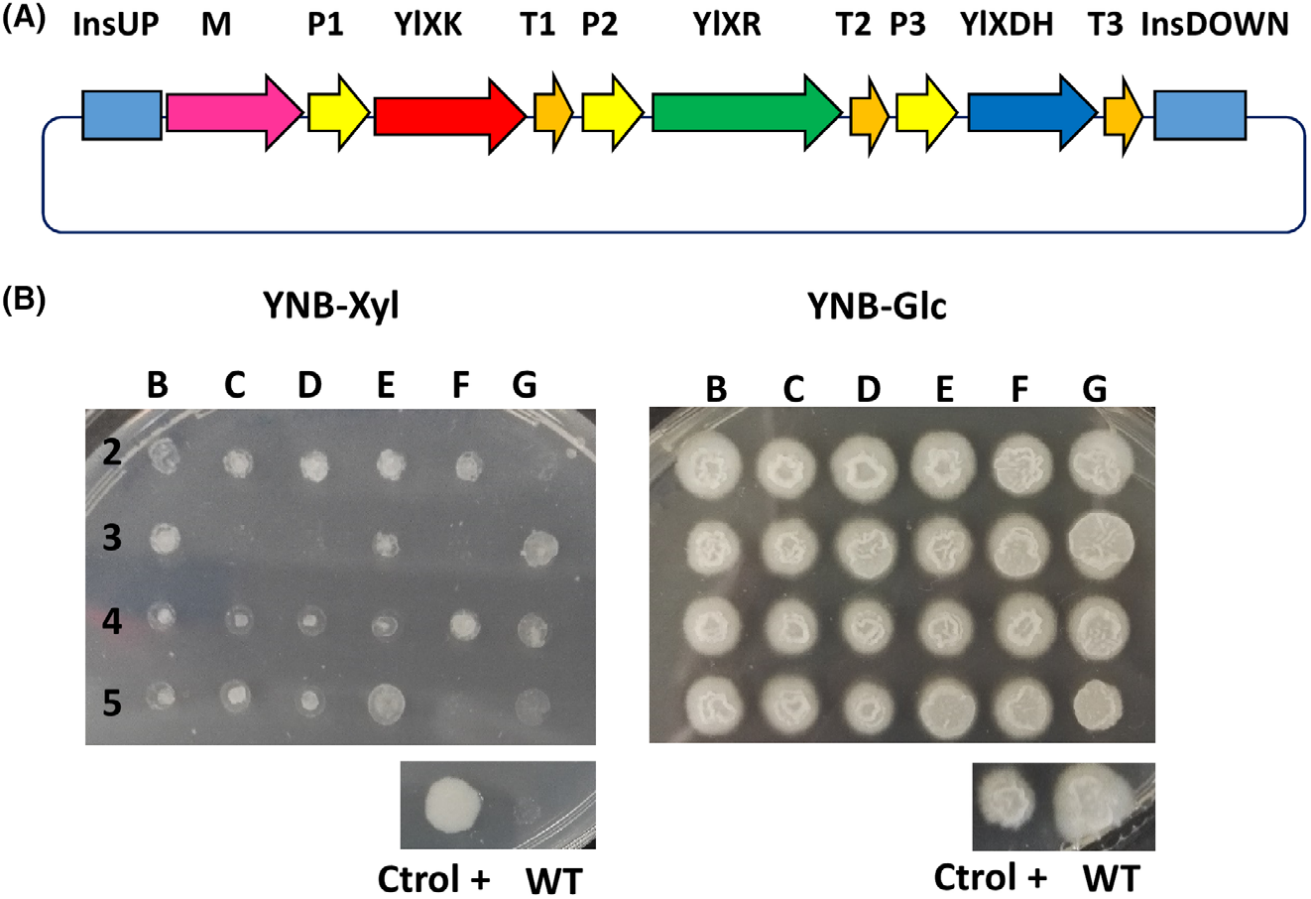
Synthetic pathway assembly using the Golden Gate toolkit and the resulting expression in *Y. lipolytica*. A. Schematic representation of a three‐gene assembly, composed of *Y. lipolytica* xylulokinase (yl*XK*), *Y. lipolytica* xylitol dehydrogenase (yl*XDH*) and *Y. lipolytica* xylose reductase (yl*XR*) genes, that allows *Y. lipolytica* to grow on xylose. B. *Y. lipolytica* clones after transformation with the xylose cassette that were grown in a plate containing only xylose as the carbon source; clones were grown in a plate containing glucose as the carbon source for the control. The xylose cassette was correctly expressed in 79% of JMY1212 transformants. WT: wild type. Xyl: xylose. Glc: glucose. Ctrol+: positive control, which was the *Y. lipolytica* strain expressing the three genes developed via the standard cloning method and whose xylose utilization was verified (Ledesma‐Amaro *et al*., 2016).

*Yarrowia lipolytica* cannot naturally use xylose even though it has genes that code for the utilization pathway. Xylose is a major component of lignocellulosic material, and microbial cell factories must be able to exploit this compound as a carbon source. To demonstrate the utility of the GG tool in this context, we assembled an overexpressed xylose utilization pathway that was based on three genes: one for xylitol dehydrogenase *XDH* (YALI0E12463g), one for xylose reductase *XR* (YALI0D07634g) and one for xylulokinase *XK* (YALIF10923g). This pathway allows *Y. lipolytica* to grow using xylose as its sole carbon source (Niehus *et al*., 2018). The three genes were assembled in a single plasmid (Fig. 6A) using the protocol we describe in the supplementary material. The strain Y1212 was then transformed with this construct, and its ability to grow on xylose was evaluated. We found that 79% of the transformants correctly expressed the xylose cassette (Fig. 6B). The overall process, from cloning to phenotype screening, took less than 10 days, which is much faster than carrying out sequential plasmid transformation, in which it is impossible to verify phenotypes before the three genes have been co‐expressed.

## Discussion

Here, we present a Golden Gate toolkit for *Y. lipolytica* that can be used to rapidly assemble multigene pathways. It is a powerful new tool for one‐step strain engineering. A colour‐based reporter system is included in the backbone vectors, which reduces the possible number of false‐positive clones during the selection process and thus speeds up assembly validation (see supplementary protocol S4). The toolkit is available to the research community through Addgene, under the plasmid ID numbers 120730‐120793, and will be regularly updated. At present, it contains 64 GG bricks: 27 promoter bricks; 14 terminator bricks; 6 markers; 10 genome‐insertion sequences corresponding to three different insertion loci and one for random integration; five gene bricks coding for fluorescent proteins that can serve as validation genes in positions 1, 2 or 3; and two destination vectors (see Fig. S1). The versatility and standardization of these bricks means that the toolkit can easily be expanded by other research teams, using our detailed protocol (see the supplementary material).

The BsaI sites used in our system are not compatible with the MoClo toolkit for *S. cerevisiae* (Lee *et al*., 2015), but expression of *S. cerevisiae* genes or optimized for *S. cerevisiae* is sometimes not functional in *Y. lipolytica* as they are genetically distant. Consequently, tools developed for *S. cerevisiae* cannot be systematically used in *Y. lipolytica*. For example, when we used the mTurquoise fluorescent protein from the *S. cerevisiae* MoClo toolkit, it was not functional in our system (data not shown). In contrast, when we used the version of mTurquoise optimized for *Y. lipolytica*, the protein was expressed correctly (Fig. 5). Likewise, we attempted to use the p*TEF* promoter from the *S. cerevisiae* MoClo toolkit when expressing RedStarII, but it was non‐functional in *Y. lipolytica* (data not shown). These results highlight the risks that bricks will be incompatible between these two hosts.

Other multigene assembly systems dedicated to *Y. lipolytica* have been described recently. The YaliBricks system (Wong *et al*., 2017) uses the BioBricks standard and four compatible restriction enzyme sites for modular pathway assembly. The tool contains 12 natural promoters, of which p*TEF* is the strongest. In this system, the use of a fluorescent reporter was unsuccessful because the signal was too weak, and luminescence had to be used instead (Wong *et al*., 2017). Our system provides a larger range of expression levels via the deployment of synthetic promoters, which are well described here (Fig. 2). An additional advantage of synthetic promoters is that they are probably much less susceptible to as‐yet‐unknown regulatory mechanisms, which is not the case for endogenous promoters of genes from the lipogenic pathway used in Wong *et al*. (2017). This problem may be especially important in oleaginous organisms. Moreover, our system seems less susceptible to the transcription inhibition that can occur when multigene constructs are under the control of the same promoter, a phenomenon observed by Wong *et al*. (2017). Another point of contrast is that, while the YaliBricks system has been used to successfully assemble five genes from the violacein pathway, it relies on sequential gene assembly. In our GG system, the use of a single restriction site means that assembly is performed in a single ‘step’. Indeed, with a one‐pot reaction, we are able to assemble three TUs, a marker and various integration site sequences, which correspond to the assembly of twelve fragments in a destination vector.

Very recently, Holkenbrink *et al*. (2018) developed a toolkit based on an alternative USER cloning approach, which allows the integration of one to two TUs at intergenic regions called EasyCloneYALI. The system largely utilizes a ku70 mutant background and a CRISPR/Cas9 strategy, and it carries out improved homologous recombination at specific non‐coding loci. However, the ku70 mutant has a low transformation rate (Verbeke *et al*., 2013), which might make it less suitable for the engineering of multiple and/or successive genes. Our system can be used with any genetic background, including wild‐type strains. The EasyCloneYALI toolkit contains 14 different promoters. However, only eight of them functionally expressed a fluorescent protein. The nine promoters provided in our GG toolkit have been tested and validated, and their expression capacity has been characterized. Very recently, Egermeier *et al*. (2019) published a GG method for *Y. lipolytica* that is based on the GoldenMOCS system. Their method systemically requires several cloning steps, which make it fundamentally different from ours. Essentially, Egermeier *et al*. have used it to produce a CRISPR/Cas9 plasmid, as well as an expression vector containing one TU, which was based on a replicative plasmid. At this stage, it is much less modular and expandable than our GG system when it comes to the expression of multigene pathways.

Here, we engineered a xylose utilization pathway to highlight how our GG toolkit can greatly accelerate the build–test step of the classical design–build–test–learn cycle in synthetic biology. It is particularly important to be able to construct multi‐TU plasmids because single genes may not yield the desired phenotype and the functionality of a single gene cannot be confirmed before successive transformations are carried out. The example presented here demonstrates that top‐performing transformants can be screened in one step. Previously, we used a smaller version of this toolkit to illustrate the assembly of an entire pathway, notably the carotenoid pathway (Celinska *et al*., 2017; Celińska *et al*., 2018; Larroude *et al*., 2018), and the construction of a set of expression cassettes containing multiple secretion signal sequences, which allows the optimization of protein secretion (Celińska *et al*., 2018; Soudier *et al*., 2019). Here, we have greatly expanded the capacity of our system, and we now provide strong as well as inducible promoters. The toolkit also contains a large panel of promoters and terminators, which allows for high‐throughput screening. More specifically, combinatorial TUs can be randomly assembled in donor vectors, generating a pool that can be screened for the best combination. Here, we have limited ourselves to providing bricks for a subset of lipid metabolism genes that could be disrupted. Indeed, we wish to underscore that our toolkit could be easily employed as a ‘consolidated’ approach, which carries out pathway integration and gene deletion simultaneously. However, in theory, any integration site sequences could be used. The goal is for users to be able to delete the targets of their choice, and our GG toolkit allows them to quickly and easily design and assemble cassettes that function simultaneously in disruption and overexpression.

## Experimental procedures

### Strains and media

The *Y. lipolytica* strains constructed and used in this study are listed in Table S1, as are the *E. coli* strains that hosted the GG‐assembled vectors.

*Escherichia coli* strain DH5α was used for cloning and plasmid propagation. The transformation of chemically competent *E. coli* cells was performed using a heat shock protocol. Cells were grown at 37°C with constant shaking on 5 ml of LB medium (10 g l^−1^ tryptone, 5 g l^−1^ yeast extract and 10 g l^−1^ NaCl); ampicillin (100 μg ml^−1^) or kanamycin (50 μg ml^−1^) was added for plasmid selection.

*Yarrowia lipolytica* strain JMY1212 strain was used in this study. The YNBD minimal media contained 10 g l^−1^ glucose (Sigma‐Aldrich, Saint‐Quentin Fallavier, France), 1.7 g l^−1^ yeast nitrogen base (YNBww; Difco, Paris, France), 5.0 g l^−1^ NH4Cl and 50 mM phosphate buffer (pH 6.8). Glucose was replaced with erythritol (10 g l^−1^) in the induction experiments or with xylose (2% wt/vol) for the purposes of verifying the xyl+ phenotypes. When necessary, the YNB medium was supplemented with uracil (0.1 g l^−1^) or leucine (0.1 g l^−1^). Solid media for *E. coli* and *Y. lipolytica* were prepared by adding 15 g l^−1^ agar (Invitrogen, Saint‐Aubin, France) to liquid media.

### Building the brick plasmids

Primers carrying predesigned 4‐nt overhangs and externally located BsaI recognition sites were created and used to amplify the bricks, which were cloned in donor vectors (Zero Blunt^®^ TOPO^®^ PCR Cloning Kit, Thermo Fisher, UK) unless otherwise stated. They were then transformed into *E. coli* (see the Data S1). PCR amplifications were performed using Q5 high‐fidelity DNA polymerase (NEB) or GoTaq DNA polymerase (Promega). The native sequences used as building bricks were amplified from the genome of *Y. lipolytica* W29. When needed, PCR fragments were purified using the QIAquick Gel Extraction Kit (Qiagen, Hilden, Germany). The mTurquoise gene (Lee *et al*., 2015) was codon optimized for *Y. lipolytica* using COOL online software (Chin *et al*., 2014). The *Streptomyces noursei nat1* gene (conferring nourseothricin resistance), the *E. coli hph* gene (conferring hygromycin resistance) (Tsakraklides *et al*., 2018), the *RedStarII* gene and the *YFP* gene were adapted for the GG system by eliminating internal BsaI recognition sites and adding the corresponding overhangs. They were synthesized by Twist Bioscience and then cloned into a TOPO vector. The genes *nat1* and *hph* were already codon optimized for *Y. lipolytica*, and their corresponding bricks contained p*TEF* and T*Lip2* for expression in *Y. lipolytica*. T*Synth8* and T*Guo* (Curran *et al*., 2015) were synthetized by annealing single‐strand sequences, and they were then cloned into a TOPO vector.

All donor vectors were verified by restriction profile analysis (BsaI) and by sequencing. All restriction enzymes were purchased from New England Biolabs (NEB), and sequencing was carried out by Eurofins Genomics. The primer sequences used in this study for DNA module construction can be found in the Data S1, and the complete list of plasmids is available in Table S1. The sequences of all the building bricks can be found in File S1. The genes in the xylose pathway were synthesized and adapted for the GG system by eliminating internal BsaI recognition sites and adding the corresponding overhangs; they were then cloned in TOPO or pUC57 vectors, giving rise to GGV pUC57 G1 *XDHno*‐BsaI, GGV pUC57 G2 *XRno*‐BsaI and GGE 0097 TOPO G3 *XKno‐*BsaI. The three genes were assembled using the GG protocol (see the Data S1), giving rise to plasmid named GGE0106 (ZUpNotI_*URA*_P1*Tef*_*XDH*_T1*Lip2*_P2T*ef*_*XR*_T2*Lip2*_P3*Tef*_*XK*_T3*Lip2*_ZDNotI).

### GG cloning procedures

Plasmids from *E. coli* were extracted using the QIAprep Spin Miniprep Kit (Qiagen). All the reactions were performed according to the manufacturer's instructions. The plasmids containing the building bricks and the destination vector (flanked with the BsaI site and predesigned overhangs) were added in equimolar quantities (50 pmoles of each DNA fragment to be assembled) to a one‐pot reaction together with 5 U of BsaI (NEB), 200 U of T4 ligase (NEB), 2 μl of T4 DNA ligase buffer (NEB) and up to 20 μl of ddH_2_O. The following thermal programme was used: [37°C for 5 min, 16°C for 2 min] × 60, 55°C for 5 min, 80°C for 5 min and 15°C ∞. Afterwards, 10 μl of the reaction mixture was used for *E. coli* transformation. White colonies were screened for correct GG assembly by colony PCR; plasmid isolation and restriction digestion with NotI were then performed for verification. The detailed protocol is described in the supplementary material. Overall, when 1 to 2 TUs were assembled (corresponding to 7–10 fragments), 50% of the *E. coli* clones showed the white phenotype, and 90% of them displayed correct assembly. When three TUs were assembled (corresponding to 13 fragments), 20% of the *E. coli* clones showed the white phenotype, and 80% of them displayed correct assembly.

To improve assembly efficiency for the three TUs, the procedure was split into two parts: creation of preassembly constructs and assembly of the multigene construct. During preassembly, we carried out three separate reactions with four GG parts each (InsertionSiteUp‐Marker‐Promoter1‐Gene1, Terminator1‐Promoter2‐Gene2‐Terminator2 and Promoter3‐Gene3‐Terminator3‐InsertionSIteDown). All the parts were present in equimolar quantities (50 pmoles). Each reaction also included 5 U of BsaI, 200 U of T4 ligase, 1 μl of T4 DNA ligase buffer (NEB) and up to 10 μl of ddH_2_O. A short thermal programme was used: [37°C for 3 min, 16°C for 2 min] × 30, 55°C for 5 min, 80°C for 5 min and 15°C ∞. During the assembly of the multigene construct, the three previous reactions were mixed together in the same tube along with the destination vector (50 pmoles), 20 U of BsaI, 400 U of T4 ligase, 4 μl of T4 DNA ligase buffer (NEB) and up to 40 μl of ddH_2_O. The following thermal programme was used: [37°C for 5 min, 16°C for 5 min] × 50, 55°C for 5 min, 80°C for 5 min and 15°C ∞. Subsequently, 15 μl of the GG reaction was transformed into *E. coli* and plated in a selective medium. White colonies were screened for correct GG assembly by colony PCR. Plasmid isolation and restriction digestion with NotI were performed for verification. When this method was employed, the rate of correct assembly was similar to that obtained for 1–2 TUs.

### Construction of *Y. lipolytica* strains

Correct GG assemblies were subsequently linearized using the NotI restriction enzyme to allow the release of the expression cassette, and 10 μl was used to transform *Y. lipolytica* JMY1212, yielding prototrophic transformants. Transformation was performed using the lithium‐acetate method (Le Dall *et al*., 1994), and transformants were selected using YNB medium. To screen for antibiotic resistance, transformation reactions were plated on YPD containing hygromycin (200 μg ml^−1^) or nourseothricin (500 μg ml^−1^).

### Growth and fluorescence analysis

*Yarrowia lipolytica* precultures were grown for 24 h in YNBD medium (supplemented with uracil when needed) in 96‐well plates. Two μl was then transferred into 200 μl of fresh medium in 96‐well microplates (OD_600nm_ of 0.1). YNB medium, supplemented with glucose (10 g l^−1^, YNBD) or erythritol (10 g l^−1^, YNBE), was used in the growth and fluorescence analysis (i.e. the choice depended on the promoters). The growth analysis was performed using a microtitre plate reader (Synergy Mx; BioTek, Colmar, France) in accordance with the manufacturer's instructions; the settings were 28°C and constant shaking. Then, every 30 min for 72 h, OD_600nm_ as well as red, yellow and blue fluorescence was measured. The wavelength settings (excitation/emission) were 558 nm/586 nm, 505 nm/530 nm and 435 nm/478 nm for red, yellow and blue fluorescence respectively. Fluorescence was expressed as mean specific fluorescence values (SFU/h, mean value of SFU per hour). Cultures were performed at least in duplicate.

### Screening for Lip2 deletion

To screen strains for the ∆lip2 phenotype, isolated transformants were grown on 200 μl of YNB for 12 h in a 96‐well plate (one colony per well); a drop test was then carried out using a YNB‐tributyrin plate supplemented with 10 g l^−1^ of tributyrin. The stock solution of tributyrin (20% tributyltin, 1% Tween) was subjected to 1 min of sonication three times on ice to obtain an emulsion. Colonies and halos could be observed after 2–3 days of culture at 28°C.

### Screening for gsy1 deletion

To screen strains for the ∆gsy phenotype, isolated transformants were grown on 200 μl of YPD for 12 h in a 96‐well plate (one colony per well). The plate was then centrifuged for 5 min at 570 × *g*. The supernatant was eliminated, and 30 μl of Lugol's solution was added to the plate to stain the pellet. The difference between negatives and positives was visible within 1–2 min. Lugol's solution was prepared by mixing solutions of 2% KI and 1% I_2_ in a ratio of 1:1.

### Screening for xylose utilization

To screen for strains expressing the xylose pathway, isolated transformants were grown in YNB agar plates containing 2% xylose as the sole carbon source. We were able to identify positive colonies after 2–3 days of growth at 28°C.

## Conflict of interest

The authors declare that they have no competing interests.

## Supporting information


**Fig. S1.** Schematic representation of all the bricks available for each position with the corresponding 4‐nt overhangs.

**Table S1.** List of strains and plasmids.

**File S1.** Fasta sequences of each DNA module.

**Data S1.** Protocol for assembling a multigene pathway using the Golden Gate toolkit for *Yarrowia lipolytica*.
