# Supplementary figures and images for: A modular Golden Gate toolkit for Yarrowia lipolytica synthetic biology
Source: Microb Biotechnol. 2019 May 31;12(6):1249–59. doi: 10.1111/1751-7915.13427 (PMC6801146; doi:10.1111/1751-7915.13427)

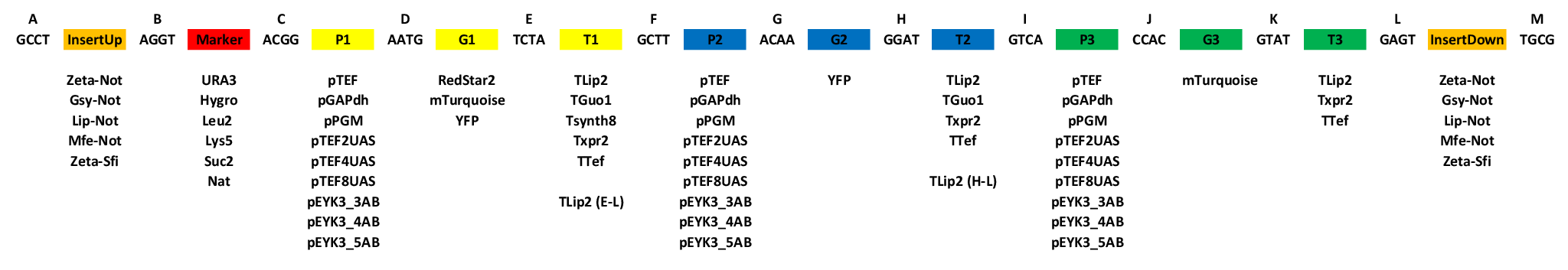

Supplement: Supplementary file 1 — Fig. S1. Schematic representation of all the bricks available for each position with the corresponding 4‐nt overhangs. [file MBT2-12-1249-s001.tif]
