# Supplementary material for: A modular Golden Gate toolkit for Yarrowia lipolytica synthetic biology
Source: Microb Biotechnol. 2019 May 31;12(6):1249–59. doi: 10.1111/1751-7915.13427 (PMC6801146; doi:10.1111/1751-7915.13427)
Supplement: Supplementary file 2 — Table S1. List of strains and plasmids. [file MBT2-12-1249-s002.docx]

Supplementary Table 1. List of strains and plasmids

| Strains | | *Genotype/plasmid* | | references |  |
| --- | --- | --- | --- | --- | --- |
| *Y. lipolytica* | |  | |  |  |
| W29 | | *Wild type* | | (Barth and Gaillardin, 1996) |  |
| JMY195, Po1d | | *MATA ura3-302 leu2‐270 xpr2‐322* | | (Barth and Gaillardin, 1996) |  |
| JMY1212 | | Po1d *Δlip2 Δlip7 Δlip8*-*LEU2-*ZETA | | (Emond, et al., 2010) |  |
| JMY5219 | | JMY1212 lys5::URA3ex | | unpublished |  |
| JMY1350 | | JMY1212-URA3 | | This study |  |
| JMY7621 | | JMY1212 pTEF-RedstarII-Tlip-*URA3* ex | | (Park, et al., 2018) |  |
| JMY7622 | | JMY1212 pGAP-RedstarII-Tlip-*URA3* ex | | This study |  |
| JMY7623 | | JMY1212 pPGM-RedstarII-Tlip-*URA3* ex | | This study |  |
| JMY7624 | | JMY1212 pTEF-2UAS-RedstarII-Tlip-*URA3* ex | | This study |  |
| JMY7625 | | JMY1212 pTEF-4UAS-RedstarII-Tlip-*URA3* ex | | This study |  |
| JMY7626 | | JMY1212 pTEF-8UAS-RedstarII-Tlip-*URA3* ex | | This study |  |
| JMY7382 | | JMY1212 pEYK1-RedstarII-Tlip-*URA3* ex | | (Park, et al., 2018) |  |
| JMY7627 | | JMY1212 pEYK1-2AB-RedstarII-Tlip-*URA3* ex | | (Park, et al., 2018) |  |
| JMY7345 | | JMY1212 pEYK1-3AB-RedstarII-Tlip-*URA3* ex | | (Park, et al., 2018) |  |
| JMY7628 | | JMY1212 pEYK1-4AB-RedstarII-Tlip-*URA3* ex | | (Park, et al., 2018) |  |
| JMY7390 | | JMY1212 pEYK1-5AB-RedstarII-Tlip-*URA3* ex | | (Park, et al., 2018) |  |
| JMY7253 | | JMY1212 pTEF-Turquoise-Tlip-*URA3* ex | | This study |  |
| JMY7652 | | JMY1212 pTEF-YFP-Tlip-*URA3* ex | | This study |  |
| JMY7653 | | JMY1212 pTEF-RedStar-TLip-pTEF-YFP-TLip-pTEF-Turquoise-Tlip-*URA3* ex | | This study |  |
| JMY7655 | | Po1d pTEF-RedstarII-Tlip-*LEU2* | | This study |  |
| JMY7656 | | JMY5219 pTEF-RedstarII-Tlip-*Lys5* | | This study |  |
| JMY7657 | | JMY1212 pTEF-RedstarII-Tlip-*hph* | | This study |  |
| JMY7658 | | JMY1212 pTEF-RedstarII-Tlip-*nat* | | This study |  |
| JMY7659 | | JMY1212 pTEF-RedstarII-TTef-pEYK2AB-YFP-Tlip-*URA3* ex | | This study |  |
| JMY7660 | | JMY1212 pTEF-RedstarII-TXpr2- pEYK2AB-YFP-Tlip-*URA3* ex | | This study |  |
| JMY7661 | | JMY1212 pTEF-RedstarII-TGuo- pEYK2AB-YFP-Tlip-*URA3* ex | | This study |  |
| JMY7662 | | JMY1212 pTEF-RedstarII-TSynth8- pEYK2AB-YFP-Tlip-*URA3* ex | | This study |  |
| JMY7663 | | JMY1212 pTEF-RedstarII-Tlip- pEYK2AB-YFP-Tlip-*URA3* ex | | This study |  |
| JMY5738 | | XYL+ Obese strain. | | (Ledesma-Amaro, et al., 2016) |  |
| JMY7767 | | Y1212 + GGA Xyl-Ura | | This study |  |
|  | |  | |  |  |
| *E. coli* | | *Genotype/plasmid* | | references | Addgene ID number |
| DH5**α** | | Φ80*lacZ*Δm15 Δ(*lac*ZYA-*arg*F) U169 *rec*A1 *end*A1 *hsd*R17 (r_k_^−^, m_k_^+^) *pho*A *sup*E44 *thi*-1 *gyr*A96 *rel*A1 λ^-^ | | (Promega) |  |
| GGE029 | pSB1A3-GB3 | | (Celinska, et al., 2017) | | 120730 |
| GGE114 | pSB1A3-ZetaUP-URA3-RFP-ZetaDOWN | | (Celinska, et al., 2017) | | 120731 |
| GGE083 | pCR4Blunt-TOPO-M-URA3 | | (Celinska, et al., 2017) | | 120732 |
| GGE176 | pCR4Blunt-TOPO-M-LYS5 | | (Celinska, et al., 2017) | | 120733 |
| GGE105 | pCR4Blunt-TOPO-M-LEU2 | | This work | | 120734 |
| GGE142 | pCR4Blunt-TOPO-M-Suc2 | | This work | | 120735 |
| GGE367 | pCR4Blunt-TOPO-M-hph | | This work | | 120736 |
| GGE368 | pCR4Blunt-TOPO-M-Nat | | This work | | 120737 |
| GGE085 | pCR4Blunt-TOPO-P1 pTEF | | (Celinska, et al., 2017) | | 120738 |
| GGE002 | pCR4Blunt-TOPO-P1 pGAPdh | | (Celinska, et al., 2017) | | 120739 |
| GGE001 | pCR4Blunt-TOPO- P1 pPGM | | (Celinska, et al., 2017) | | 120740 |
| GGE145 | pCR4Blunt-TOPO- P1 TEF-2UAS | | (Celinska, et al., 2017) | | 120741 |
| GGE146 | pCR4Blunt-TOPO- P1 TEF-4UAS | | (Celinska, et al., 2017) | | 120742 |
| GGE147 | pCR4Blunt-TOPO- P1 TEF-8UAS | | (Celinska, et al., 2017) | | 120743 |
| GGE0104 | pUC57 - P1 pEYK1-3AB | | (Park, et al., 2018) | | 120744 |
| GGE0132 | pUC57 - P1 pEYK1-4AB | | (Park, et al., 2018) | | 120745 |
| GGE250 | pUC57 - P1 pEYK1-5AB | | (Park, et al., 2018) | | 120746 |
| GGE006 | pCR4Blunt-TOPO-P2 pTEF | | (Celinska, et al., 2017) | | 120747 |
| GGE005 | pCR4Blunt-TOPO-P2 pGAPdh | | (Celinska, et al., 2017) | | 120748 |
| GGE004 | pCR4Blunt-TOPO- P2 pPGM | | (Celinska, et al., 2017) | | 120749 |
| GGE150 | pCR4Blunt-TOPO- P2 TEF-2UAS | | (Celinska, et al., 2017) | | 120750 |
| GGE151 | pCR4Blunt-TOPO- P2 TEF-4UAS | | (Celinska, et al., 2017) | | 120751 |
| GGE152 | pCR4Blunt-TOPO- P2 TEF-8UAS | | (Celinska, et al., 2017) | | 120752 |
| GGE108 | pCR4Blunt-TOPO- P2 pEYK1-3AB | | This work | | 120753 |
| GGE139 | pCR4Blunt-TOPO- P2 pEYK1-4AB | | This work | | 120754 |
| GGE129 | pCR4Blunt-TOPO- P2 pEYK1-5AB | | This work | | 120755 |
| GGE009 | pCR4Blunt-TOPO-P3 pTEF | | (Celinska, et al., 2017) | | 120756 |
| GGE008 | pCR4Blunt-TOPO-P3 pGAPdh | | (Celinska, et al., 2017) | | 120757 |
| GGE007 | pCR4Blunt-TOPO-P3 pPGM | | (Celinska, et al., 2017) | | 120758 |
| GGE292 | pCR4Blunt-TOPO-P3 TEF-2UAS | | (Celinska, et al., 2017) | | 120759 |
| GGE294 | pCR4Blunt-TOPO-P3 TEF-4UAS | | (Celinska, et al., 2017) | | 120760 |
| GGE317 | pCR4Blunt-TOPO-P3 TEF-8UAS | | (Celinska, et al., 2017) | | 120761 |
| GGE273 | pCR4Blunt-TOPO-P3 pEYK1-3AB | | This work | | 120762 |
| GGE275 | pCR4Blunt-TOPO-P3 pEYK1-4AB | | This work | | 120763 |
| GGE259 | pCR4Blunt-TOPO-P3 pEYK1-5AB | | This work | | 120764 |
| GGE014 | pCR4Blunt-TOPO-T1 Lip2 | | (Celinska, et al., 2017) | | 120765 |
| GGE177 | pCR4Blunt-TOPO-T1 XPR2 | | (Celinska, et al., 2017) | | 120766 |
| GGE082 | pCR4Blunt-TOPO-T1 TEF | | (Celinska, et al., 2017) | | 120767 |
| GGE025 | pCR4Blunt-TOPO-T1 SynthGuo | | (Celinska, et al., 2017) | | 120768 |
| GGE027 | pCR4Blunt-TOPO-T1 Synth8 | | (Celinska, et al., 2017) | | 120769 |
| GGE015 | pCR4Blunt-TOPO-T2 Lip2 | | (Celinska, et al., 2017) | | 120770 |
| GGE257 | pCR4Blunt-TOPO-T2 XPR2 | | This work | | 120771 |
| GGE256 | pCR4Blunt-TOPO-T2 TEF | | This work | | 120772 |
| GGE260 | pCR4Blunt-TOPO-T2 SynthGuo | | This work | | 120773 |
| GGE080 | pCR4Blunt-TOPO-T3 Lip2 | | (Celinska, et al., 2017) | | 120774 |
| GGE258 | pCR4Blunt-TOPO-T3 XPR2 | | This work | | 120775 |
| GGE286 | pCR4Blunt-TOPO-T3 TEF | | This work | | 120776 |
| GGE020 | pCR4Blunt-TOPO-TLip2 (E-L) | | (Celinska, et al., 2017) | | 120777 |
| GGE 021 | pCR4Blunt-TOPO-TLip2 (H-L) | | (Celinska, et al., 2017) | | 120778 |
| GGE077 | pCR4Blunt-TOPO-G1-RedStarII | | This work | | 120779 |
| GGE270 | pCR4Blunt-TOPO-G1-YFP | | This work | | 120780 |
| GGE190 | pUC-G1-mTurquoise | | This work (Twist) | | 120781 |
| GGE070 | pCR4Blunt-TOPO-G2-YFP | | (Celinska, et al., 2017) | | 120782 |
| GGE261 | pCR4Blunt-TOPO-G3-mTurquoise | | This work | | 120783 |
| GGE067 | pCR4Blunt-TOPO-Zeta-NotI_Up | | (Celinska, et al., 2017) | | 120784 |
| GGE038 | pCR4Blunt-TOPO-Zeta-NotI_Down | | (Celinska, et al., 2017) | | 120785 |
| GGE091 | pCR4Blunt-TOPO-Zeta-SfiI_Up | | (Celinska, et al., 2017) | | 120786 |
| GGE094 | pCR4Blunt-TOPO-Zeta-SfiI_Down | | (Celinska, et al., 2017) | | 120787 |
| GGE255 | pCR4Blunt-TOPO-Lip2-NotI_Up | | This work | | 120788 |
| GGE254 | pCR4Blunt-TOPO-Lip2-NotI_Down | | This work | | 120789 |
| GGE253 | pCR4Blunt-TOPO-Gsy-NotI_Up | | This work | | 120790 |
| GGE252 | pCR4Blunt-TOPO-Gsy-NotI_Down | | This work | | 120791 |
| GGE207 | pCR4Blunt-TOPO-MFE-NotI_Up | | This work | | 120792 |
| GGE209 | pCR4Blunt-TOPO-MFE-NotI_Down | | This work | | 120793 |
