# Supplementary material for: A modular Golden Gate toolkit for Yarrowia lipolytica synthetic biology
Source: Microb Biotechnol. 2019 May 31;12(6):1249–59. doi: 10.1111/1751-7915.13427 (PMC6801146; doi:10.1111/1751-7915.13427)
Supplement: Supplementary file 4 — Data S1. Protocol for assembling a multigene pathway using the Golden Gate toolkit for Yarrowia lipolytica. [file MBT2-12-1249-s004.docx]

# Protocol for assembling a multigene pathway using the Golden Gate toolkit for *Yarrowia lipolytica*

[Protocol for assembling a multigene pathway using the Golden Gate toolkit for *Yarrowia lipolytica* 1](#_Toc3371038)

[1.1 Introduction 1](#_Toc3371039)

[1. Materials 2](#_Toc3371040)

[1.2 Construction of GG vectors 2](#_Toc3371041)

[1.2.1 Construction of donor vectors with GG parts 2](#_Toc3371042)

[1.2.2 Assembly of expression vectors 3](#_Toc3371043)

[1.3 Transformation into a *Y. lipolytica* strain 5](#_Toc3371044)

[1.3.1 Expression cassette preparation 5](#_Toc3371045)

## Introduction

The Golden Gate (GG) toolkit for *Y. lipolytica* is a modular cloning approach, based on BsaI type IIS restriction enzymes, for assembling multiple genes via a single-step, one-pot reaction (Fig. 1). Type II enzymes cut outside their recognition sites to excise sequences with arbitrarily defined four-base overhangs. The DNA modules are assembled on a scaffold of predesigned 4-nucleotide (nt) overhangs that cover three transcription units (each bearing a promoter, gene, and terminator), a selective marker, target sequences for genome integration, and a destination vector backbone, thus constituting thirteen elements in total (Fig. 1).


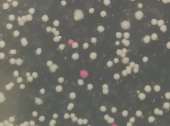

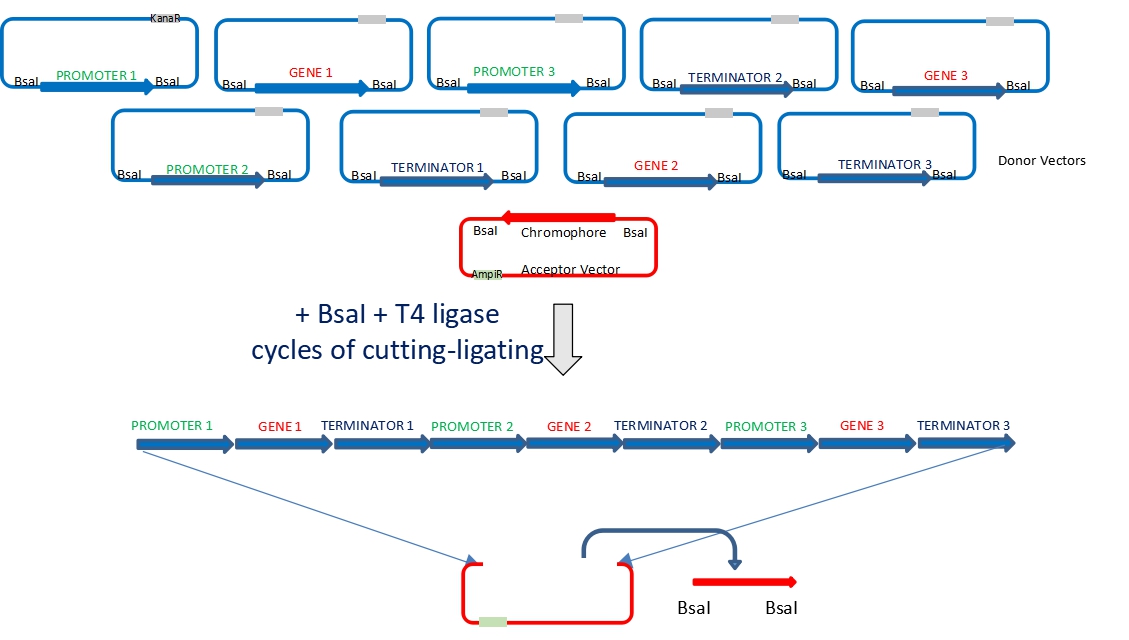


Figure 1. Schematic diagram of the Golden Gate assembly process

## Materials

1. BsaI restriction enzyme (10,000 U/mL) (NEB)
2. T4 ligase (400,000 U/ml) (NEB)
3. T4 ligase buffer (NEB)
4. NotI (R3189L; NEB)
5. Q5 High-Fidelity 2x Master Mix (M0492L; NEB)
6. *E. coli* competent cells
7. LB medium
8. Selective plates (LBagar-ampicillin and LBagar-kanamycin)
9. YNB selective media for yeast
10. YPD rich media
11. Thermocycler
12. Plasmid extraction kit (Macherey-Nagel)
13. Destination vector
14. pCRII-BluntTOPO vector (Invitrogen)
15. 28°C, 37°C, and 42°C water baths; incubators
16. Lithium acetate (FLUKA), PEG4000 (BDH), single-strand salmon sperm DNA (Sigma) or

Frozen-EZ Yeast Kit (Zymo research) for yeast transformation

## Construction of GG vectors

### Construction of donor vectors with GG parts

1. The DNA to be used for the GG parts should be screened for the presence of internal BsaI sites. If there are any, they should be eliminated. To do so, a point mutation can be performed, taking care not to change the amino acid sequence and to preferentially use codons that are highly common in *Y. lipolytica*. This task can be carried out directly by ordering synthetic DNA from specialized companies or by performing site-directed mutagenesis via PCR.
2. Amplify the DNA to be used for the GG parts by PCR. Based on the position of the GG part, assign the corresponding 4-nt overhangs to be added to the primers. Descriptions of the primer sequences and design, with the 4-nt overhang, are provided in Table 1. Primers should have at least 20-nt overlaps with the sequence to be amplified and a Tm of 60-65°C. The external BsaI site with the appropriate 4-nt overhang is added to the 5' end of each DNA strand.

| Position | Fw primer | Rv primer | 5' overhang | 3' overhang |
| --- | --- | --- | --- | --- |
| InsertUp (NotI) | gggGGTCTCt**GCCT***GCGGCCGC*nnn | cccGGTCTCt**ACCT**nnn | **GCCT** | **AGGT** |
| Marker | gggGGTCTCt**AGGT**nnn | cccGGTCTCt**CCGT**nnn | **AGGT** | **ACGG** |
| Promoter1 | gggGGTCTCt**ACGG**nnn | cccGGTCTCt**CATT**nnn | **ACGG** | **AATG** |
| Gene1 | gggGGTCTCt**AATG**nnn | cccGGTCTCt**TAGA**nnn | **AATG** | **TCTA** |
| Terminator1 | gggGGTCTCt**TCTA**nnn | cccGGTCTCt**AAGC**nnn | **TCTA** | **GCTT** |
| Promoter2 | gggGGTCTCt**GCTT**nnn | cccGGTCTCt**TTGT**nnn | **GCTT** | **ACAA** |
| Gene2 | gggGGTCTCt**ACAA**nnn | cccGGTCTCt**ATCC**nnn | **ACAA** | **GGAT** |
| Terminator2 | gggGGTCTCt**GGAT**nnn | cccGGTCTCt**TGAC**nnn | **GGAT** | **GTCA** |
| Promoter3 | gggGGTCTCt**GTCA**nnn | cccGGTCTCt**GTGG**nnn | **GTCA** | **CCAC** |
| Gene3 | gggGGTCTCt**CCAC**nnn | cccGGTCTCt**ATAC**nnn | **CCAC** | **GTAT** |
| Terminator3 | gggGGTCTCt**GTAT**nnn | cccGGTCTCt**ACTC**nnn | **GTAT** | **GAGT** |
| InsertDown (NotI) | gggGGTCTCt**GAGT**nnn | cccGGTCTCt**CGCA***GCGGCCGC*nnn | **GAGT** | **TGCG** |
| Destination vector | gggGGTCTCt**TGCG**nnn | cccGGTCTCt**AGGC**nnn | **TGCG** | **GCCT** |

Table 1. Primer design for each position. Underlined sequences: BsaI recognition site. **Bold sequences**: overhangs generated after digestion. *Italic sequences*: NotI recognition site. n: corresponds to the overlap of at least 20 nt with the sequence to be amplified.

1. Amplify the DNA of interest with primers that add both the BsaI recognition site and the specific 4-nt overhang; use a high-fidelity polymerase.
2. Purify the PCR-amplified DNA either directly from the reaction or from an agarose gel if there are any non-specific amplification products; use a commercial kit (Macherey-Nagel) according to the manufacturer’s instructions.
3. Clone the PCR fragment into the donor vector with a backbone bearing the kanamycin-resistance gene (e.g.,Zero Blunt® TOPO® PCR Cloning Kit (ThermoFisher)) according to the manufacturer’s instructions.
4. Transform the donor vectors into *E. coli* competent cells and grow them on LB-kanamycin plates.
5. Verify isolated colonies by colony PCR using M13 standard primers. If you are using the Zero Blunt® TOPO® PCR Cloning Kit, the product should have the size of the insert + 244 bp on the agarose gel.
6. Grow PCR-positive colonies overnight in 5 mL of LB with 50 µg/mL of kanamycin and perform plasmid preparation using a commercial Miniprep kit.
7. Verify the sequence of the insert.
8. Store positive clones at –80°C in glycerol stock.

All the GG parts that are already available (e.g., promoters, terminators, markers) and their corresponding donor vectors are listed in the main article.

### Assembly of expression vectors

1. Prepare all the GG parts (donor vectors) and the destination vector (GGE029 or GGE114 with ampicillin-resistance gene). Extract the plasmids from the *E. coli* strains containing the donor and destination vectors using a commercial Miniprep Kit in accordance with the manufacturer’s instructions. The concentration of all plasmid preparations should be determined using Nanodrop or an equivalent technique.
2. Prepare the GG assembly reaction:

Mix together all the GG parts needed and the destination vector in equimolar quantities (50 pmol). Add 1 µl BsaI + 1 µl T4 ligase + 2 µl T4 ligase buffer + up to 20 µl of ddH_2_O.

Place the reaction in a thermocycler, and use the following thermal program:

(37°C for 5 min; 16°C for 5 min) x 50

37°C for 10 min

80°C for 5 min

15°C ∞

1. To improve efficiency when assembling **three TUs**, the procedure can be split into two parts: the creation of preassembly constructs and the assembly of the multigene construct.

Preassembly:

1. Prepare three separate reactions with four GG parts each. All parts are represented in equimolar quantities (50 pmoles).
2. InsertionSiteUp + Marker + Promoter1 + Gene1
3. Terminator1 + Promoter2 + Gene2 + Terminator2
4. Promoter3 + Gene3 + Terminator3 + InsertionSiteDown.
5. Add to each reaction 0.5 µl BsaI + 0.5 µl T4 ligase + 1 µl T4 ligase buffer + up to 10 µl of ddH_2_O.
6. Place each reaction in a thermocycler, and run the following thermal program:

(37°C for 3 min, 16°C for 2 min) x 30

55°C for 5 min

80°C for 5 min

15°C ∞

Assembly of multigene construct:

1. Mix together the three previous reactions (1,2,3) in the same tube, and add the following:

10 µl Rxn1 + 10 µl Rxn2 + 10 µl Rxn3 + 50 pmoles destination vector + 2 µl BsaI + 2 µl T4 ligase + 4 µl T4 ligase buffer + up to 40 µl of ddH_2_O

1. Place the mixture in a thermocycler, and run the following thermal program:

(37°C for 5 min, 16°C for 5 min) x 50

55°C for 5 min

80°C for 5 min

15°C ∞

1. Transform the result in *E. coli* DH5a competent cells by adding 10 µl of the assembly reaction (15 µl when the 2-step procedure is performed) to 80 µl of competent cells, and then plate the cells on LB agar + 100 µg/mL ampicillin.
2. Verify isolated white colonies by colony PCR with the appropriate primers (i.e., depending on the assembly). The expression vector contains an RFP chromophore drop-out cassette to allow for colony selection (red/white) prior to colony screening for correct assembly (see Fig. 1).
3. Grow PCR-positive colonies overnight in 5 mL LB+50 µg/mL kanamycin, and then extract the plasmid using a commercial Miniprep kit.
4. Carry out NotI digestion to verify the digestion profile.
5. Store positive clones at –80°C in glycerol stock.

## Transformation into a *Y. lipolytica* strain

### Expression cassette preparation

1. Extract the plasmid of interest using a Miniprep commercial kit.
2. Digest the expression vector to release the expression cassette (most commonly with NotI digestion or SfiI).
3. Transform *Y. lipolytica* using the lithium-acetate method as described in (Barth and Gaillardin 1996). Alternatively, use the Frozen-EZ Yeast Transformation Kit (Zymo research), and plate the result on YNB selective media (i.e., choose based on your marker).
